# P09 Feasibility of retrospective chart review to assess alignment of urinary tract infection diagnosis, testing and treatment decisions with UKHSA diagnostic guidance in patients 65 years+ in the emergency department

**DOI:** 10.1093/jacamr/dlad077.013

**Published:** 2023-08-02

**Authors:** Mandy Slatter, Alastair Hay, Matthew Jones

**Affiliations:** Royal United Hospitals Bath NHS Trust, Bath, UK; University of Bristol, Bristol, UK; Department of Life Sciences, University of Bath, Bath, UK

## Abstract

**Background:**

UKHSA urinary tract infection (UTI) diagnostic guidance uses presence/absence of specific symptoms/signs to guide decisions on urine culture and antibiotic treatment.^1^ The goal is treatment of those with highest risk of infection, avoiding serious illness, whilst limiting antibiotic overuse. UK improvement initiatives^2,3^ encourage hospitals to align with UKHSA guidance. UTI is a common infection in the emergency department (ED) and diagnosis can be challenging in the elderly.^4,5^

**Objectives:**

To explore retrospective chart review to quantify UTI treatment alignment with UKHSA UTI guidance.^1^ Identify ED patients aged 65 years+ (65+) with primary diagnosis of UTI. Limit to non-admitted for manageable sample. Complete retrospective chart review to elicit documented UTI symptoms/signs and associated: (i) urine dipstick testing; (ii) urine sampling for microscopy, culture and susceptibility (MC&S); and (iii) antibiotic treatment. Assess alignment with relevant UKHSA guidance. Estimate time taken.

**Methods:**

Electronic patient record search for key terms (Table 1) identified 6076 ED attendances 65+ between 1 August and 31 October 2021. Forty patients met inclusion criteria. Paramedic/ED notes were reviewed, and information gathered regarding presence/absence of: UTI symptoms/signs as per UKHSA guidelines;^1^ urine dipstick test; urine for MC&S; and UTI antibiotic treatment. Findings were mapped to illustrate if UKHSA diagnostic pathway and intended antibiotic prescribing decisions followed.

**Table 1.** Key terms

Search terms	Search fields
Urin, UTI, pyelonephritis, cystitis, urosepsis, CAUTI, AKI, Acute Kidney Injury, Flank pain	Reason for visit, Chief Complaint, Presenting Complaint, ECDS

**Results:**

Twenty-seven of 40 patients matched UKHSA criteria for lower UTI/pyelonephritis. Three of 27 followed recommended pathway of: no dipstick; urine for MC&S; and antibiotic. Twenty of 27 had urine dipstick test (not recommended); 16/27 had urine sample for MC&S (recommended); 26/27 received an antibiotic (recommended). Twenty-three who received antibiotics had dipstick testing, no urine for MC&S or both. One patient did not receive indicated antibiotic treatment. Documented symptoms/signs did not indicate UTI/pyelonephritis in 13/40 patients. Two of 13 followed the recommended pathway of: no dipstick; no urine for MC&S; and no antibiotic. Seven of 13 received a dipstick test; 6/13 had urine for MC&S; 9/13 received antibiotics which were not indicated. Five of 40 followed the recommended pathway; 30/40 received the recommended treatment. The time cost was around 30 min/patient.

**Conclusions:**

UKHSA guideline alignment was low. Testing in asymptomatic patients appeared to result in unnecessary antibiotic use. Retrospective chart review generated detailed data to assess alignment, however results may represent poor documentation not poor alignment. Retrospective chart review time cost too high for large numbers and assumes accurate documentation.

## References

[1] Urinary Tract Infection: Diagnostic Tools for Primary Care. GOV.UK. https://www.gov.uk/government/publications/urinary-tract-infection-diagnosis

[2] Antimicrobial Resistance CQUIN 2019/20. NHS England. https://www.england.nhs.uk/antimicrobial-resistance-cquin-2019-20/

[3] Commissioning for Quality and Innovation (CQUIN) Scheme for 2022/23 Annex: Indicator Specifications. NHS England. https://www.england.nhs.uk/publication/combined-ccg-icb-and-pss-commissioning-for-quality-and-innovation-cquin-indicator-specification/

[4] Goebel MC . The five Ds of antibiotic stewardship for urinary tract infection. Clin Microbiol Rev 2021; 34: e0000320. 10.1128/CMR.00003-20 PMC8404614 34431702

[5] Rowe TA . Diagnosis and management of urinary tract infection in older adults. Infect Dis Clin North Am 2014; 28: 75–89. 24484576 10.1016/j.idc.2013.10.004 PMC4079031

